# Supplementary material for: Nanoscale spatial dependence of avidity in an IgG1 antibody
Source: Sci Rep. 2021 Jun 16;11:12663. doi: 10.1038/s41598-021-92280-2 (PMC8209022; doi:10.1038/s41598-021-92280-2)
Supplement: Supplementary file 1 — Supplementary Informations. [file 41598_2021_92280_MOESM1_ESM.pdf]

Supplementary information for:

Nanoscale spatial dependence of avidity in an IgG1 antibody

Agnieszka Jendroszek,[1,2] and Magnus Kjaergaard[1-4*]

[1] Department of Molecular Biology and Genetics, Aarhus University

[2] The Danish Research Institute for Translational Neuroscience (DANDRITE)

[3] Aarhus Institute of Advanced Studies (AIAS)

[4] The Center for Proteins in Memory (PROMEMO)

[*] Corresponding author: magnus@mbg.au.dk

Sequences of APH constructs

Color code:

His-tag Thrombin site *Flexible linker*

APH$_{half}$

MGSS**HHHHHH**SSGMKQLEKELKQLEKELQAIEKQLAQLQKKAQARKKKLAQLKKKLQA*PGSGSGP*MKQLEKELKQLEKELQAIEKQLAQLQWKAQARKKKLAQLKKKLQA

APH$_{6}$

MGSS**HHHHHH**SSGMKQLEKELKQLEKELQAIEKQLAQLQWKAQARKKKLAQLKKKLQA

APH$_{6_thrombin}$

MGSS**HHHHHH**SSGLVPRGSHMKQLEKELKQLEKELQAIEKQLAQLQWKAQARKKKLAQLKKKLQA

APH$_{8}$

MGSS**HHHHHH**SSGMKQLEKELKQLEKELQAIEKQLAQLQKKLQAIEKQLAQLQWKAQARKKKLAQLKKKLQA

APH$_{8_thrombin}$

MGSS**HHHHHH**SSGLVPRGSHMKQLEKELKQLEKELQAIEKQLAQLQKKLQAIEKQLAQLQWKAQARKKKLAQLKKKLQA

APH$_{10}$

MGSS**HHHHHH**SSGMKQLEKELKQLEKELQAIEKQLAQLQKKAQARKKKLKQLEKELQAIEKQLAQLQWKAQARKKKLAQLKKKLQA

APH$_{10_thrombin}$

MGSS**HHHHHH**SSGLVPRGSHMKQLEKELKQLEKELQAIEKQLAQLQKKAQARKKKLKQLEKELQAIEKQLAQLQWKAQARKKKLAQLKKKLQA

APH$_{12}$

MGSSHHHHHHSSGMKQLEKELKQLEKELQAIEKQLAQLQKKAQARKKKLAQLKKKLQALEKELKQLEKE
LQAIEKQLAQLQWKAQARKKKLAQLKKKLQA

APH$_{12_thrombin}$

MGSSHHHHHHSSGLVPRGSHMKQLEKELKQLEKELQAIEKQLAQLQKKAQARKKKLAQLKKKLQALEKE
LKQLEKELQAIEKQLAQLQWKAQARKKKLAQLKKKLQA

APH$_{16}$

MGSSHHHHHHSSGMKQLEKELKQLEKELQAIEKQLAQLQKKAQARKKKLQALEKELKQLEKELQAIEKQL
AQLQKKAQARKKKLAQLKKKLKQLEKELQAIEKQLAQLQWKAQARKKKLAQLKKKLQA

APH$_{16_thrombin}$

MGSSHHHHHHSSGLVPRGSHMKQLEKELKQLEKELQAIEKQLAQLQKKAQARKKKLQALEKELKQLEKEL
QAIEKQLAQLQKKAQARKKKLAQLKKKLKQLEKELQAIEKQLAQLQWKAQARKKKLAQLKKKLQA

APH$_{18}$

MGSSHHHHHHSSGMKQLEKELKQLEKELQAIEKQLAQLQKKAQARKKKLAQLKKKLQALEKELKQLEKE
LQAIEKQLAQLQKKAQARKKKLAQLKKKLQALEKELKQLEKELQAIEKQLAQLQWKAQARKKKLAQLKKK
LQA

APH$_{18_thrombin}$

MGSSHHHHHHSSGLVPRGSHMKQLEKELKQLEKELQAIEKQLAQLQKKAQARKKKLAQLKKKLQALEKE
LKQLEKELQAIEKQLAQLQKKAQARKKKLAQLKKKLQALEKELKQLEKELQAIEKQLAQLQWKAQARKKK
LAQLKKKLQA

APH$_{24}$

MGSSHHHHHHSSGMKQLEKELKQLEKELQAIEKQLAQLQKKAQARKKKLAQLKKKLQALEKELKQLEKE
LQAIEKQLAQLQWKAQARKKKLAQLKKKLQALEKELKQLEKELQAIEKQLAQLQKKAQARKKKLAQLKKK
LQALEKELKQLEKELQAIEKQLAQLQWKAQARKKKLAQLKKKLQA

APH$_{24_thrombin}$

MGSSHHHHHHSSGLVPRGSMKQLEKELKQLEKELQAIEKQLAQLQKKAQARKKKLAQLKKKLQALEKELK
QLEKELQAIEKQLAQLQWKAQARKKKLAQLKKKLQALEKELKQLEKELQAIEKQLAQLQKKAQARKKKLA
QLKKKLQALEKELKQLEKELQAIEKQLAQLQWKAQARKKKLAQLKKKLQA

Table S1. Kinetic constants determined by SPR for APH variants binding to THE His Ab.

pH		BIACORE evaluation software				IGOR Pro	
		k_{on1} $10^3 \times (Ms)^{-1}$	k_{off1} (ms^{-1})	k_{on2} $10^{-4} \times (Ms)^{-1}$	k_{off2} (ms^{-1})	k_{off1} (ms^{-1})	k_{off2} (ms^{-1})
5.8	APH_6	95.8 ± 0.5	18.5 ± 0.2	2.74 ± 0.02	0.80 ± 0.01	22.6 ± 1.3	1.1 ± 0.12
	APH_8	76.7 ± 0.4	13.7 ± 0.2	1.63 ± 0.02	0.63 ± 0.02	18.9 ± 1.0	0.78 ± 0.10
	APH_{10}	122.5 ± 0.2	14.8 ± 0.1	2.39 ± 0.01	0.66 ± 0.01	19.5 ± 1.1	0.70 ± 0.068
	APH_{12}	100.8 ± 0.4	19.9 ± 0.2	1.74 ± 0.01	0.62 ± 0.01	19.6 ± 1.1	0.60 ± 0.050
	APH_{16}	29.0 ± 0.3	20.8 ± 0.6	0.94 ± 0.02	0.77 ± 0.03	16.7 ± 1.4	0.74 ± 0.14
	APH_{18}	47.7 ± 0.3	19.5 ± 0.3	1.16 ± 0.01	1.26 ± 0.02	20.9 ± 1.7	1.5 ± 0.16
	APH_{24}	65.2 ± 0.4	21.4 ± 0.4	1.08 ± 0.01	1.48 ± 0.02	17.0 ± 1.2	1.3 ± 0.15
	APH_{half}	54.6 ± 1.4	18.5 ± 0.1	n.d.	n.d.	n.d.	n.d.
6.0	APH_{12}	38.7 ± 0.3	4.30 ± 0.13	0.54 ± 0.01	0.18 ± 0.003	6.5 ± 0.6	0.17 ± 0.0135
	APH_{half}	118.8 ± 0.5	8.61 ± 0.06	n.d.	n.d.	n.d.	n.d.
6.2	APH_{12}	66.9 ± 0.1	3.76 ± 0.02	0.72 ± 0.004	0.11 ± 0.001	3.8 ± 0.3	0.074 ± 0.0085
	APH_{half}	152.7 ± 0.7	6.22 ± 0.02	n.d.	n.d.	n.d.	n.d.

Errors represent s.d. from three experiments from the BIACORE evaluation software, and as detailed on Fig. S7 for IGOR PRO analysis.

Table S2. Molecular weight of APH variants calculated from the sequence of the monomer ($MW_{sequence\_monomer}$) and determined by SAXS (MW_{SAXS}).

	**$MW_{sequence\_monomer}$ (kDa)**	**MW_{SAXS} (kDa)**
APH_6	5.7	10.0
APH_8	7.3	17.0
APH_{10}	9.0	18.0
APH_{12}	10.6	21.0
APH_{16}	13.9	34.0
APH_{18}	15.5	41.0
APH_{24}	20.4	37.0

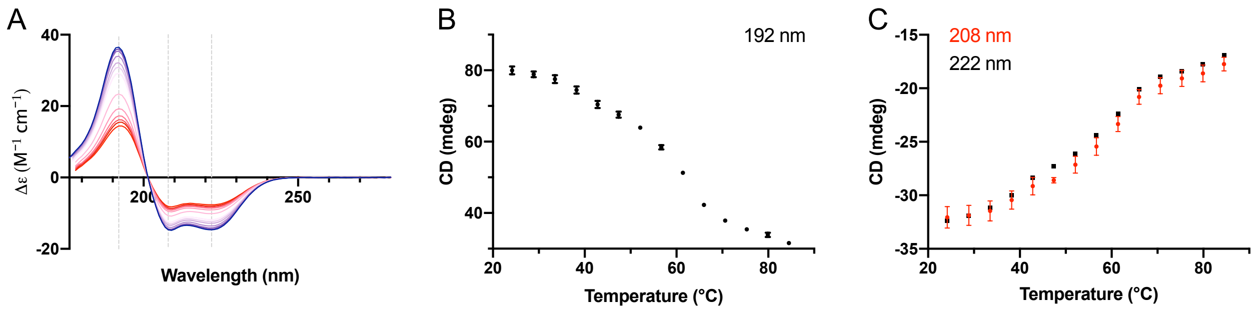

Figure S1. (A) SR-CD spectra of APH_{10} were measured at temperatures varying from 25 °C to 85 °C Blue = cold, red = hot). Dotted lines indicate the wavelength at which change of the CD signal intensity with temperature was compared in (B) and (C). Data are averaged over a 3 nm window. Error bars are the S.D. of the averaged three datapoints.

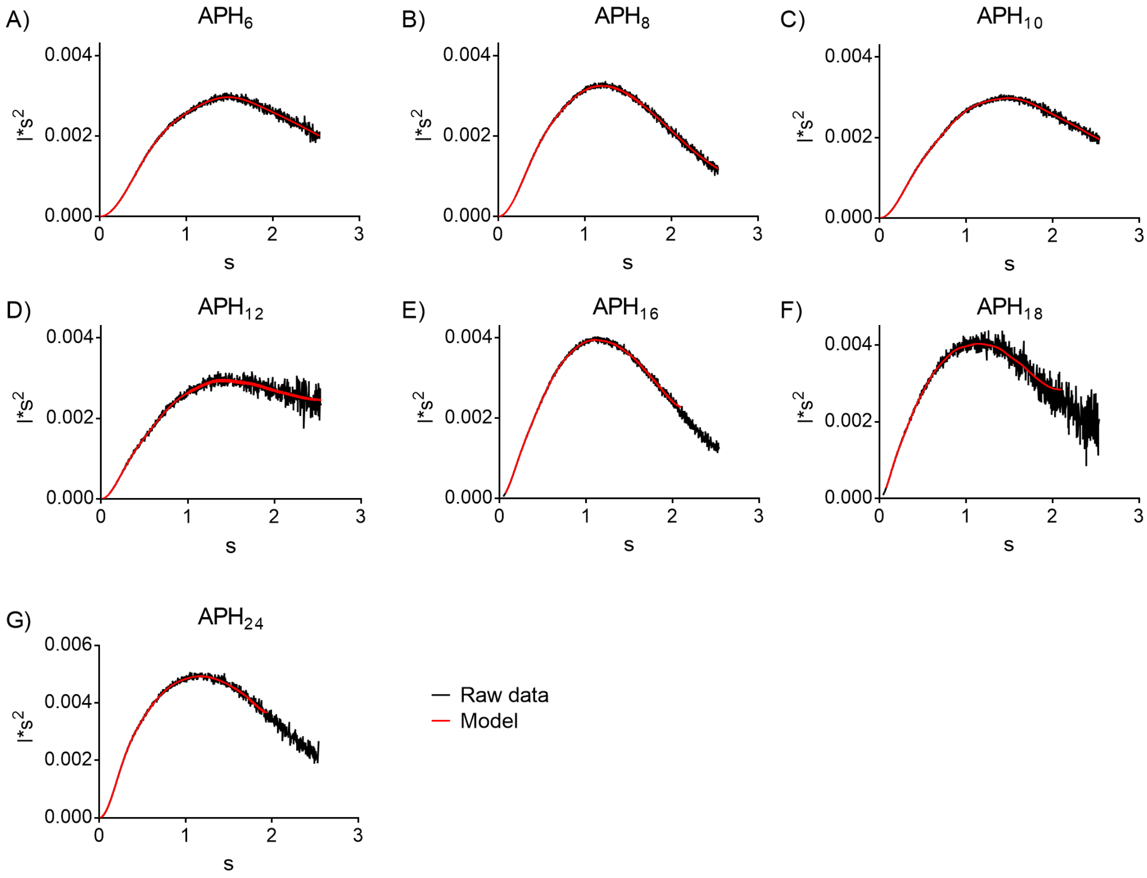

Figure S2. SAXS data of all APH variants represented as Kratky plots. Scattering back-predicted from the *ab initio* models represented a red line.

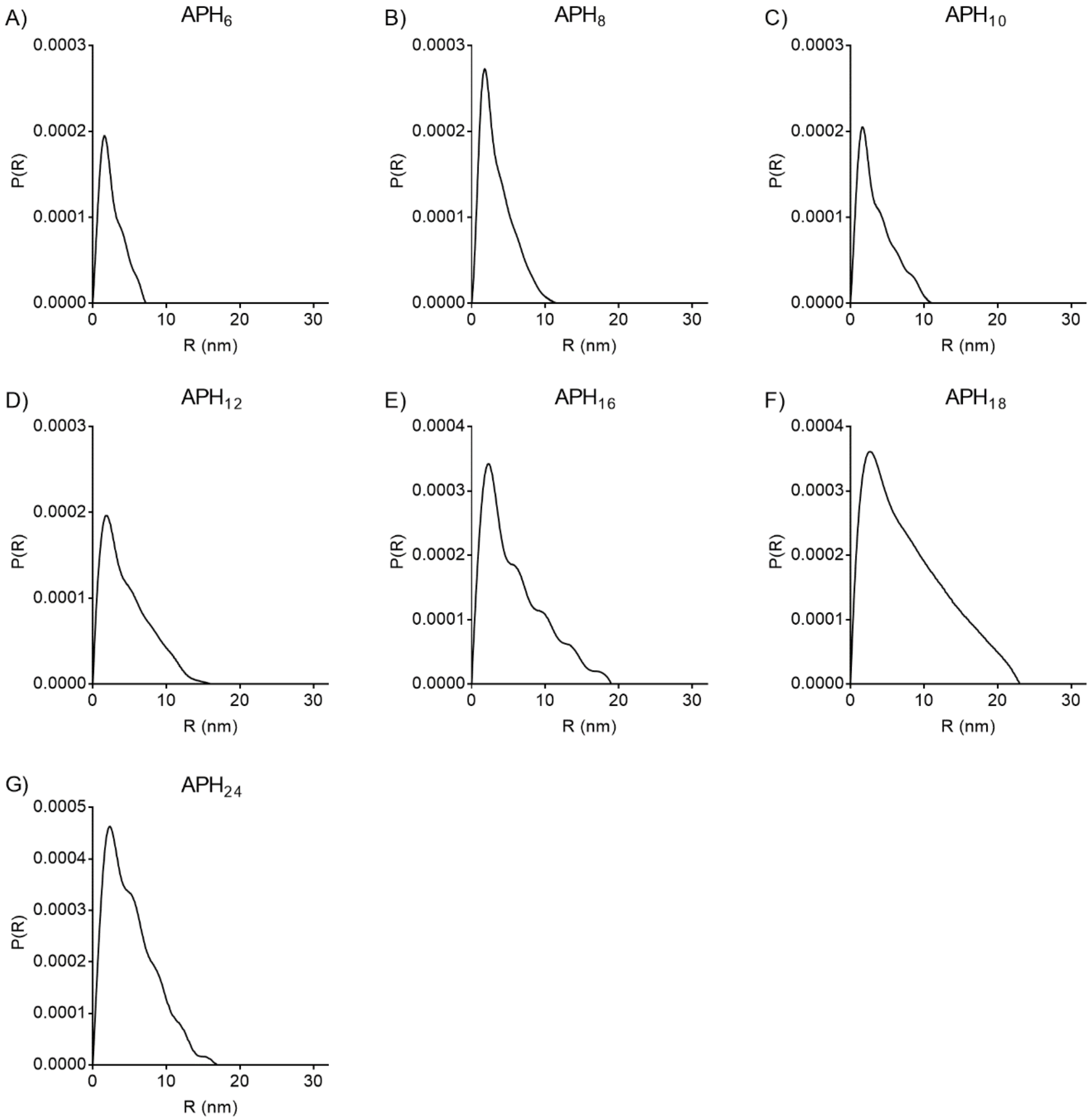

Figure S3. SAXS data for all variants represented as p(r) functions. The maximum distance (D_{max}) increases steadily up to APH_{18}, whereafter it dramatically increase in APH_{20} and is reduced in APH_{24}. These data mirror the R_g and the R_h determined from DLS.

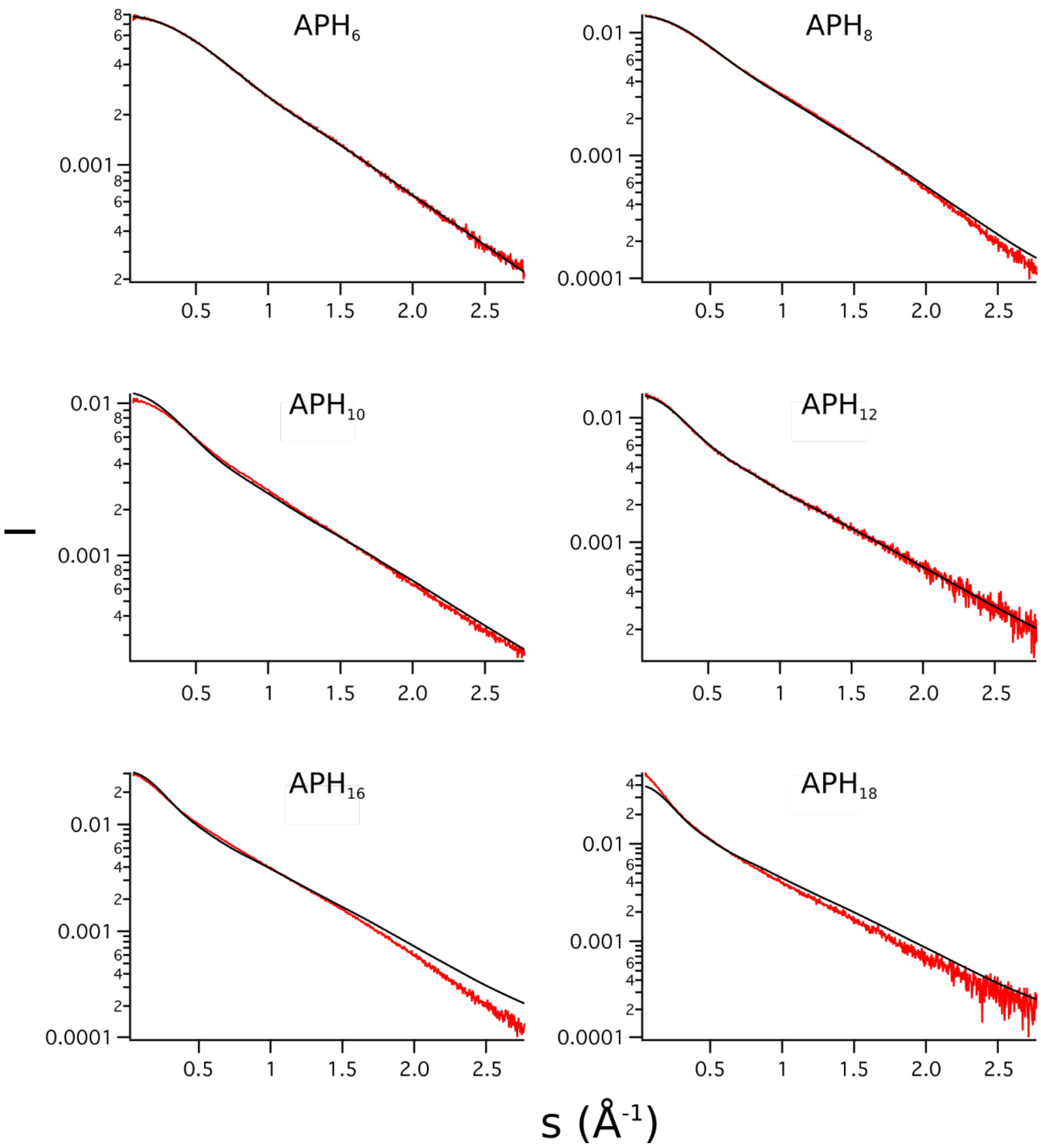

Figure S4. Comparison of experimental SAXS measurements (red) to back-predicted scattering curves from idealized coiled-coil models (black).

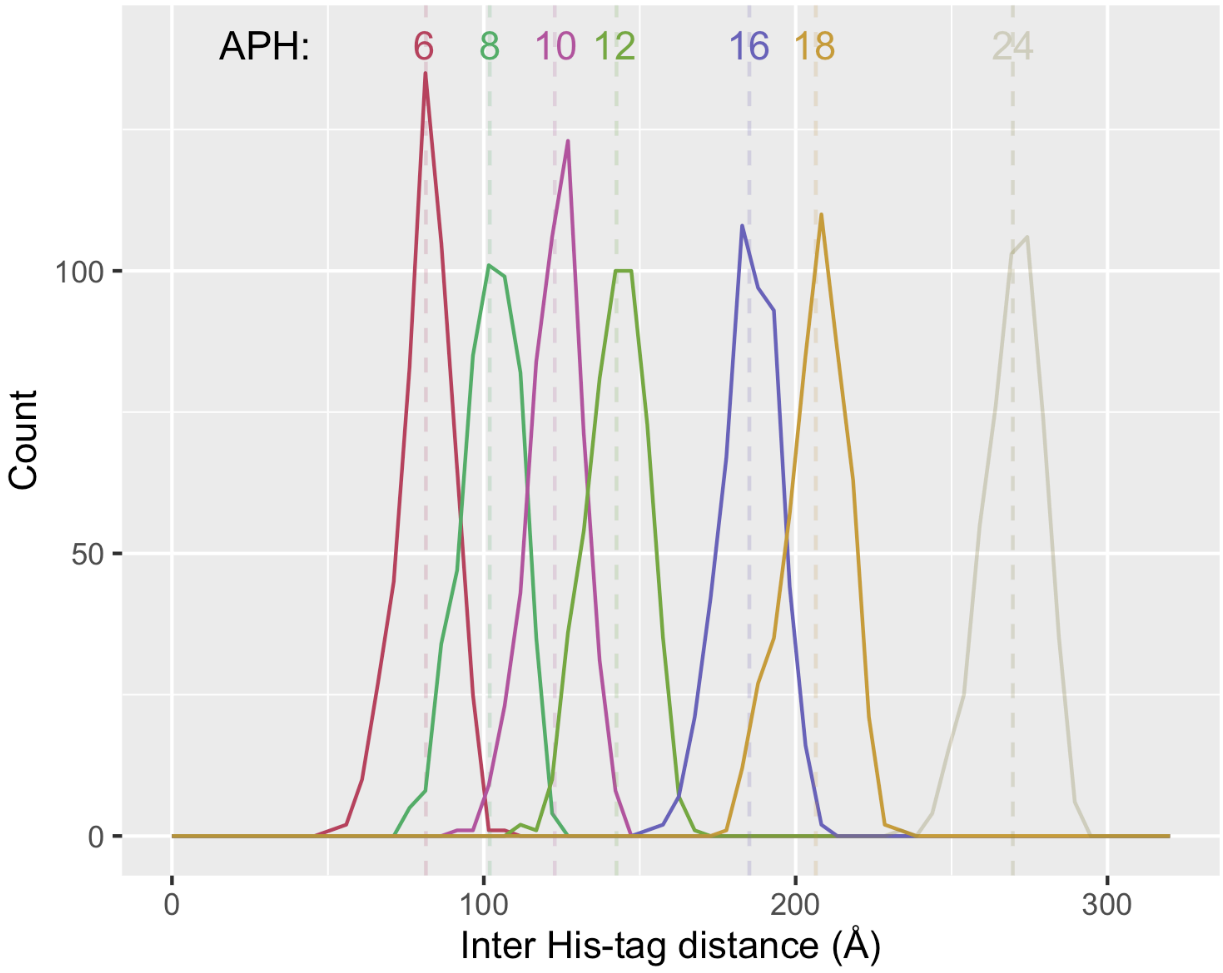

Fig. S5: Modelled inter-epitope distances in APH nanocalipers. An ensemble representing the missing residues were modelled with his-tag in 500 random coil conformations. The inter-epitope distance is measured between the third histidine in each tag. The histogram uses 5 Å bins and the mean distance shown with dashes. Nanocalipers that did not form the desired structure are translucent.

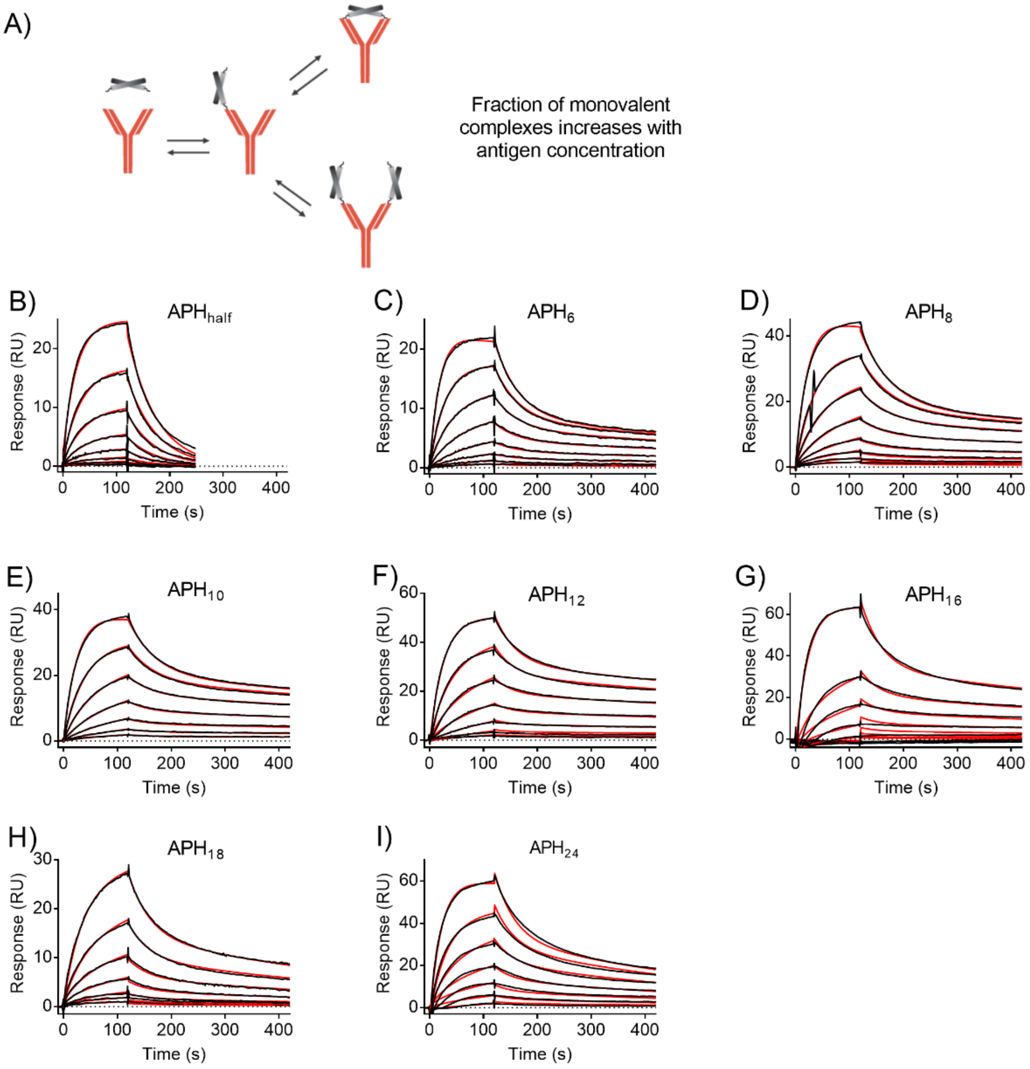

Figure S6. Binding of a bivalent antigen to an antibody can result in formation of mono- and bivalent complexes (A). Fraction of antigens interacting with an antibody via single epitope increases with the antigen concertation. Representative sensorgrams of all antigens binding to anti-His antibody at pH 5.8 are shown in (B) to (I). Black lines refer to raw data and 1:1 (APH_{half}) or bivalent analyte (APH_6, APH_{10}, APH_{12}, APH_{16}, APH_{18}, APH_{24}) fit is shown as red lines.

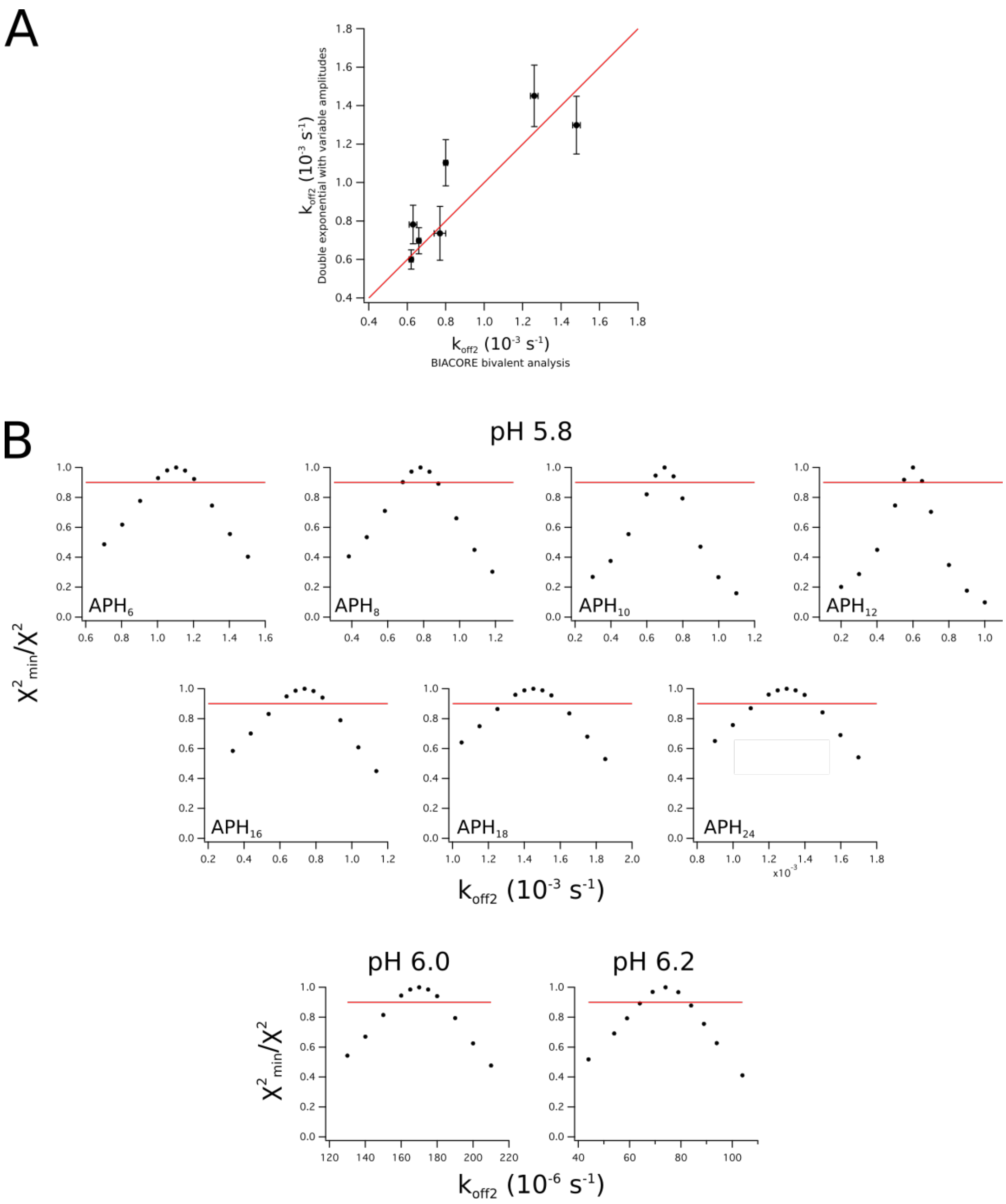

Figure S7. Confidence analysis of dissociation rate constants. A) The SPR data were either analysed by fitting the full data set to bivalent model in the BIACORE Evaluation software, resulting in adequate fits. We also fit the dissociation phase to a double exponential, where the amplitudes vary freely but the two rate constants are fitted globally, which improves the fit. B) To evaluate the confidence interval of the fitted rate constants, we evaluated the χ^2-curves using the bivalent fit by locking k_{off2} to values around the fitted value and fitting the remaining parameters. All the fitted parameters are located in well-defined χ^2 minima, suggesting they are well determined by the data. Built-in error estimations typically underestimate the error associated with co-variance between fitted variables, and therefore we determined the confidence interval from a threshold of $\chi^2_{min} / \chi^2 = 0.9$ (red line) following the criteria defined by Johnson et al. (Ref. 41 in main manuscript)
